# Fluoroquinolone Use in Food Animals

**DOI:** 10.3201/eid1111.040630

**Published:** 2005-11

**Authors:** Peter Collignon

**Affiliations:** * Canberra Hospital, Garran, Australian Capital Territory, Australia

**To the Editor:** Two recent articles (*1**,**2*) show that fluoroquinolone use in food animals is associated with infections by antimicrobial drug–resistant strains of *Campylobacter* in humans. These infections cause problems in treating illnesses as well as increased rates of illness and death (*3*). Despite a large body of scientific evidence and a judicial review (*1**–**3*) that show harmful results in many persons, some members of the poultry and pharmaceutical industries argue that fluoroquinolone use in food animals has no adverse effects in humans (*4*) and continue to supply these drugs for use in poultry (*2**,**5*). The use of these drugs has caused rapidly increasing resistance rates in most countries. In the United States, 19% of *Campylobacter* isolates from humans are now ciprofloxacin resistant (*2*), and resistance rates >80% are seen in Spain (*5*). By contrast, in Australia, where fluoroquinolones were never approved for use in food animals, domestically acquired infections with fluoroquinolone-resistant *Campylobacter* spp. are rarely found in humans (*6*). Drug-resistant *Escherichia coli* is also of concern. In Spain, humans frequently acquire fluoroquinolone-resistant *E. coli* associated with fluoroquinolone use in poultry (*7*).

In the United States, better controls in meat and poultry slaughter and processing, as well as improved food-safety education campaigns, have resulted in 28% fewer *Campylobacter* infections annually since 1996 (*8*). However, ≈1.8 million persons (600 per 100,000) are likely to contract symptomatic *Campylobacter* infections per year (*3**,**8*), and fluoroquinolone resistance is now 19% (*2*). Thus, the risk of a person's contracting fluoroquinolone-resistant *Campylobacter* infection is 114 per 100,000 per year. If 80% of *Campylobacter* infections are foodborne (*3*), and 90% of these infections are acquired from poultry (*9*), then ≈82 of 100,000 persons per will contract ciprofloxacin-resistant *Campylobacter* infections from poultry each year. Most persons with *Campylobacter* infections would not benefit from antimicrobial drug therapy. However, if only 10% of infected persons would benefit from antimicrobial drug therapy, fluoroquinolone use in poultry could cause ≈82 persons per million to have a compromised response to therapy. In the United States (population 300 million), this number translates to >24,000 persons annually.

Data on the number of animals that receive fluoroquinolones are difficult to find. Bayer (manufacturer of the only fluoroquinolone used in poultry in the United States) states that Baytril (enrofloxacin) is used in <1% of US broiler flocks (*4*). This statistic allows us to estimate how many persons will potentially have an adverse outcome compared to the number of animals receiving fluoroquinolones. If 24,000 persons in the United States have an adverse outcome annually after <84 million chickens (1% of 8.4 billion) are treated with enrofloxacin, then ≈285 persons are at risk of having an adverse outcome for every 1 million chickens treated.

This risk seems needless. In Australia, consequences from not using these agents in food animals (i.e., neither therapeutic nor prophylactic use is approved) have not been seen. Thus, I do not agree with Iovine and Blaser (*1*), who would allow fluoroquinolones to be used to treat sick food production animals. Bayer claims that "Baytril is used for therapeutic purposes only..." (*4*). Thus, continuation of fluoroquinolone use for these therapeutic purposes will allow the consequent development of resistant bacteria in humans, which will include resistant strains of *Campylobacter*, *E. coli*, and *Salmonella*. Discontinuing fluoroquinolone use by mass dosing (the current practice for poultry [10]) would decrease the amount of the drug used. However, why use fluoroquinolones at all? Narrower spectrum antimicrobial drugs (e.g., sulfonamides, amoxicillin) could be used to adequately treat sick animals. Surely *E. coli* drug resistance in food animals in the United States cannot be at a level that makes fluoroquinolones indispensable. If resistance levels to narrower spectrum antimicrobial drugs are at high levels, does this finding not imply that major changes concerning antimicrobial drug use in food animals are needed?

Better methods are needed to accurately estimate how many persons are negatively affected annually because of the misuse of antimicrobial drugs in food animals. Compromised therapeutic outcomes occur in many persons throughout the world because of fluoroquinolone-resistant *Campylobacter* infections (*10*). Fluoroquinolone use is not essential for food animal production or the welfare of animals. Many ways to keep animals healthy and productive exist other than treating or trying to prevent infections with the mass use of antimicrobial drugs such as fluoroquinolones.
