# Supplementary material for: Fluoroquinolone Use in Food Animals
Source: Emerg Infect Dis. 2005 Nov;11(11):1789–92. doi: 10.3201/eid1111.040630 (PMC3367372; doi:10.3201/eid1111.040630)
Supplement: Related texts

# Reply: Fluoroquinolone Use in Food Animals

**DOI:** 10.3201/eid1111.050652

**Authors:** Nicole M. Iovine, Martin J. Blaser

**Affiliations:** * New York University School of Medicine, New York, New York, USA; † Department of Veterans Affairs, New York, New York, USA

**In response:** We agree with Dr Collignon's view that using fluoroquinolones in the poultry industry imposes a "needless risk" of harm to humans by promoting the emergence of fluoroquinolone-resistant *Campylobacter* infections and consequent increased illness in humans (*1*). We support the therapeutic use of fluoroquinolones in poultry if only animals that are ill are treated (*2*). The widespread practice of adding fluoroquinolones to the drinking water of a hen house with thousands of birds, or of an entire flock, promotes the emergence of resistant *Campylobacter* strains. Unless veterinary practices limit fluoroquinolone use exclusively to sick birds, the only responsible recourse is to ban the use of fluoroquinolones in the poultry industry altogether, in agreement with the Food and Drug Administration's decision (*3*).

## References

[1] Collignon P. Fluoroquinolone use in food animals. Emerg Infect Dis. 2005;11:1789–90. 10.3201/eid1111.04063016422004PMC3367372

[2] Iovine NM, Blaser MJ. Antibiotics in animal feed and spread of resistant *Campylobacter* from poultry to humans. Emerg Infect Dis. 2004;10:1158–9.1522467110.3201/eid1006.040403PMC3323286

[3] Davidson DJ. In the matter of enrofloxacin for poultry: withdrawal of approval of Bayer Corporation's new animal drug application 1 (NADA) 140-828 (Baytril). In: FDA Docket No. 00N-1571; 2004.

# Reply: Fluoroquinolone Use in Food Animals

**DOI:** 10.3201/eid1111.051022

**Authors:** Amita Gupta, Robert V. Tauxe, Frederick J. Angulo

**Affiliations:** * Johns Hopkins University, Baltimore, Maryland, USA; † Centers for Disease Control and Prevention, Atlanta, Georgia, USA

**In response:** We thank Dr Collignon for his comments regarding the human health impact after fluoroquinolone use in food animals (*1*) Similar conclusions concerning the human health consequence of using fluoroquinolones in poultry in the United States were reached by the US Food and Drug Administration (FDA) in a quantitative risk assessment in 2000 (*1*). FDA concluded that fluoroquinolone use in poultry has resulted in the emergence and dissemination of fluoroquinolone-resistant *Campylobacter* that infects thousands of persons each year in the United States. Therefore, since 2000, FDA has sought to discontinue the use of fluoroquinolones in poultry. On July 25, 2005, FDA announced the withdrawal of fluoroquinolones for use in poultry effective as of September 12, 2005.

The debate regarding the use of antimicrobial agents in food animals and their impact on human health has been longstanding. For many years, public health officials have raised concern regarding the use of antimicrobial drugs in food animals that are of importance to human health. Industry representatives have stated that these concerns are unfounded. In our study, we found no fluoroquinolone resistance among a sample of *Campylobacter jejuni* strains collected from persons in 1990 (*2*). In 1995, fluoroquinolone use was approved in the United States for food animal use, specifically for poultry. Between 1997 and 2001, we noted a significant increase in fluoroquinolone resistance among human *Campylobacter* strains in the United States, monitored through Centers for Disease Control and Prevention surveillance (13%–19%, logistic regression odds ratio 2.4, 95% confidence interval 1.4–4.1). This finding means that despite a 31% decline in the overall incidence of *Campylobacter* infections from 1997 to 2001, the incidence of fluoroquinolone-resistant *Campylobacter* infections increased (*3**,**4*). More recently published data show that persons with fluoroquinolone-resistant infection have a longer duration of diarrhea and are more likely to have invasive disease or die than persons with fluoroquinolone-susceptible infections (*5**,**6*). These data demonstrate, as Dr Collignon indicates, the human health consequences of increasing fluoroquinolone resistance among *Campylobacter*.

*Campylobacter* is a zoonotic pathogen and most often associated with consumption of poultry. Our study found that in 1999, 10% of grocery store–purchased chickens yielded fluoroquinolone-resistant *Campylobacter* (*2*). More recently, retail food testing in 2002 performed by FDA found that 14% of retail chicken samples were contaminated by fluoroquinolone-resistant *Campylobacter* (*7*). Studies of commercial poultry flocks before, during, and after fluoroquinolone treatment found that only a small proportion of flocks had fluoroquinolone-resistant *Campylobacter* infections before fluoroquinolone treatment, but that fluoroquinolone-resistant strains quickly emerged during treatment and often persisted after treatment (*8**,**9*). As Dr Collignon describes, in Australia, fluoroquinolone-resistant *Campylobacter* strains have not been detected in domestically acquired human infections; this finding has been attributed to the fact that fluoroquinolones are not licensed for use in food animals (*10*). We agree with Dr Collignon that convincing data indicate that use of antimicrobial agents that are of human importance among food animals has an adverse human health impact and that the time has come to find alternatives that promote food-animal health while minimizing the induction of antimicrobial resistance.

## References

[1] Collignon P. Fluoroquinolone use in food animals. Emerg Infect Dis. 2005;11:1789–90. 10.3201/eid1111.04063016422004PMC3367372

[2] US Food and Drug Administration. The human health impact of fluoroquinolone-resistant *Campylobacter* attributed to the consumption of chicken. [cited 2005 Jun 23]. Available from http://www.fda.gov/cvm/documents/revisedRA.pdf

[3] US Food and Drug Administration. Final decision of the FDA commissioner. Withdrawal of approval of the new animal drug application for enrofloxacin in poultry. Rockville (MD): Center for Veterinary Medicine, US Food and Drug Administration; July 27, 2005. Docket no. 2000N-1571. [cited 2005 Sep 16]. Available from http://www.fda.gov/oc/antimicrobial/baytril.pdf

[4] Gupta A, Nelson JM, Barrett TJ, Tauxe RV, Rossiter SP, Friedman CR, Antimicrobial resistance among *Campylobacter* strains, United States, 1997–2001. Emerg Infect Dis. 2004;10:1102–9.1520706410.3201/eid1006.030635PMC3323172

[5] Nelson JM, Baker N, Theriot C, Vugia DJ, Beebe JL, Rabatsky-Ehr T, Increasing incidence of ciprofloxacin-resistant *Campylobacter*: Foodnet and NARMS 1997–2001. [cited 2005 Jun 23]. Available from http://www.cdc.gov/narms/publications/2003/Nelson_2003.pdf

[6] Helms M, Simonsen J, Olsen KE, Mølbak K. Adverse health events associated with antimicrobial drug resistance in *Campylobacter* species: a registry-based cohort study. J Infect Dis. 2005;191:1050–5. 10.1086/42845315747238

[7] Nelson JM, Smith KE, Vugia DJ, Rabatsky-Ehr T, Segler SD, Kassenborg HD, Prolonged diarrhea due to ciprofloxacin-resistant *Campylobacter* infection. J Infect Dis. 2004;190:1150–7. 10.1086/42328215319866

[8] National Antimicrobial Resistance Monitoring System for Enteric Bacteria (NARMS). NARMS retail meat annual report, 2002. Rockville (MD): Center for Veterinary Medicine, US Food and Drug Administration; 2002. [cited 2005 Jun 23]. Available from http://www.fda.gov/cvm/coversheet.htm

[9] Griggs DJ, Johnson MM, Frost JA, Humphrey T, Jorgensen F, Piddock LJ. Incidence and mechanism of ciprofloxacin resistance in *Campylobacter* spp. isolated from commercial poultry flocks in the United Kingdom before, during, and after fluoroquinolone treatment. Antimicrob Agents Chemother. 2005;49:699–707. 10.1128/AAC.49.2.699-707.200515673754PMC547197

[10] Unicomb L, Ferguson J, Riley TV, Collignon P. Fluoroquinolone resistance in *Campylobacter* absent from isolates, Australia. Emerg Infect Dis. 2003;9:1482–3.1471809910.3201/eid0911.030336PMC3035555
